# A comparative study on the efficacy of robot of stereotactic assistant and frame-assisted stereotactic drilling, drainage for intracerebral hematoma in patients with hypertensive intracerebral hemorrhage

**DOI:** 10.12669/pjms.38.7.5481

**Published:** 2022

**Authors:** Liang Liang, Xin Li, Haiqing Dong, Xin Gong, Guanpeng Wang

**Affiliations:** 1 Liang Liang, Department of Neurosurgery, Baoding No.1 Central Hospital, Baoding 071000, Hebei, China; 2 Xin Li, Department of Neurosurgery, Baoding No.1 Central Hospital, Baoding 071000, Hebei, China; 3 Haiqing Dong, Department of Neurosurgery, Baoding No.1 Central Hospital, Baoding 071000, Hebei, China; 4 Xin Gong, Department of Neurosurgery, Baoding No.1 Central Hospital, Baoding 071000, Hebei, China; 5 Guanpeng Wang, Department of Neuroscience Critical Care Unit, Baoding No.1 Central Hospital, Baoding 071000, Hebei, China

**Keywords:** Hypertensive intracerebral hemorrhage, Robot of stereotactic assistant, Stereotactic frame, Efficacy

## Abstract

**Objectives:**

To compare the clinical efficacy of robot of stereotactic assistant (ROSA) and frame-assisted stereotactic drilling and drainage for intracerebral hematoma in hypertensive intracerebral hemorrhage (HICH).

**Methods:**

A total of 142 patients with HICH treated in Baoding First Central Hospital from January 2018 to January 2020 were selected and divided into two groups using a random number table. The ROSA group was treated with a robot of stereotactic assistant, while the frame group underwent frame-assisted stereotactic drilling and drainage for intracerebral hematoma. Surgical duration, postoperative extubation time and complications were compared between the two groups. Venous blood (5 mL) was collected before and three days after surgery. The levels of inflammatory factors [tumor necrosis factor-α (TNF-α), high-sensitivity C-reactive protein (hs-CRP) and interleukin-6 (IL-6)], as well as neurological function indexes [neuron-specific enolase (NSE), nerve growth factor (NGF) and brain-derived neurotrophic factor (BDNF)] were detected by enzyme-linked immunosorbent assay.

**Results:**

The surgical duration, postoperative extubation time, and incidences of infection and postoperative rehemorrhage in the ROSA group were lower than those in the frame group (*P* < 0.05). In the ROSA group, postoperative TNF-α, hs-CRP, IL-6 and NSE levels were significantly lower while NGF and BDNF levels were higher than those in the frame group (all *P* < 0.05).

**Conclusion:**

Compared with frame-assisted stereotactic drilling and drainage for intracerebral hematoma, ROSA in HICH treatment shortens the surgical duration and postoperative extubation time, reduces the risks of infection and rehemorrhage and decreases inflammatory level, which is helpful for the recovery of neurological function.

## INTRODUCTION

Hypertensive intracerebral hemorrhage (HICH) is a brain disease caused by the rupture of intracranial arteries induced by a sharp increase in blood pressure.^1^ HICH is characterized by high short-term mortality and high disability rate. Brain injury caused by elevated intracranial pressure is an important cause of poor prognosis. At present, reducing intracranial pressure and surgical removal of hematoma is the key to the treatment of HICH.^2^ The stereotactic frame assistant system is a common medical instrument in neurosurgery, mainly to assist head stereotactic to determine the intracranial target. The removal effect of the stereotactic frame assistant system on intracerebral hematoma in HICH patients has been widely verified in the clinic.^3^ In recent years, Robot of Stereotactic Assistant (ROSA) is a new neurosurgical technique gradually applied, with the advantages of accurate positioning and convenient use.^4^ A previous comparative study on ROSA and stereotactic frame assistant system in the treatment of HICH has been reported, but it focused on the clinical efficacy.^5^ In addition to comparing the efficacy, this study also compared postoperative inflammatory level and neurological function of patients treated with two types of surgery, so as to compare the differences between the two types of surgery from the perspective of biological indexes.

## METHODS

A randomized controlled trial was used in this study. A total of 142 patients with HICH treated in Baoding First Central Hospital from January 2018 to January 2020 were selected, aged 21~79 years, average age of (56.51 ± 8.23) years.

### Ethical Approval

The study was approved by the Institutional Ethics Committee of Baoding No.1 Central Hospital on May 27, 2021 (No. [2021]068), and written informed consent was obtained from all participants.

### Inclusion criteria:

- Patients with the volume of supratentorial hematoma was ≥ 20 mL, or the volume of infratentorial hematoma was ≥ 10 mL in imaging examination;
- Patients with a history of hypertension and meeting the surgical indications;
- Patients with Glasgow Coma Scale (GCS) score ≥ 5;
- Patients or family members signing the informed consent and voluntarily participating in the study.

### Exclusion criteria:

- Cerebral hemorrhage caused by rupture of intracranial aneurysm or craniocerebral trauma;
- Brainstem hemorrhage;
- Dysfunction of the liver, kidney or other important organs.
- The 142 patients with HICH were divided into two groups using a random number table, with 71 patients in each group.

### Surgical Methods

ROSA group: The surgery was performed using the equipment produced by MedTech, France. The scalp was marked before surgery, and the scalp markers were registered successively after angiography. The surgical entrance was selected under laser guidance. After cutting open the soft tissue, a hole with a diameter of one cm was drilled. After the dura mater was cut open, the puncture needle was introduced with the aid of the mechanical arm, the hematoma was pumped, the drainage tube was placed and then the surgical channel was closed. After surgery, urokinase was injected according to the volume of residual blood. When the volume of hematoma < 5 mL, the drainage tube was removed.

### Frame group

The surgery was carried out using the stereotactic frame assistant system produced by Leibinger, Germany. The specific procedure is as follows: Wearing Fisher headframe, the hematoma was located using CT scanning, and the surgical target was determined according to the maximum dimension of hematoma. Based on the established surgical plan, a 4~5 cm longitudinal incision was made at the selected location. After drilling the skull, the dura mater was cross-cut and the puncture needle was inserted with the aid of a navigator. After reaching the target, the hematoma was pumped, the drainage tube was placed and then the surgical channel was closed. Postoperatively, urokinase was injected according to the volume of residual blood. When the volume of hematoma < 5 mL, the drainage tube was removed.

### Observation Indexes

The baseline data including gender, age, GCS score at admission, hematoma volume and time from onset to surgery in both groups were counted. Surgical duration, bleeding volume, postoperative extubation time and complications were also recorded. Venous blood (5 mL) was collected before and three days after surgery. The levels of inflammatory factors [tumor necrosis factor-α (TNF-α), high-sensitivity C-reactive protein (hs-CRP) and interleukin-6 (IL-6)], as well as neurological function indexes [neuron-specific enolase (NSE), nerve growth factor (NGF) and brain-derived neurotrophic factor (BDNF)], were detected using enzyme-linked immunosorbent assay.

### Statistical Analysis

The data were analyzed using SPSS23.0. The enumeration data were expressed as rate, and analyzed with the *χ^2^* test or Fisher’s exact probability test. The measurement data were expressed as *x̅*±*s*, and analyzed by the independent *t*-test. *P* < 0.05 was considered statistically significant.

## RESULTS

Gender, age, GCS score at admission, hematoma volume and time from onset to surgery showed no statistically significant differences between the two groups (*P* > 0.05), as shown in Table-I. The surgical duration and postoperative extubation time in the ROSA group were lower than those in the frame group (*P* < 0.05). However, no statistically significant difference was found in bleeding volume between the two groups (*P* > 0.05) (Table-II).

**Table I:** Comparison in baseline data between two groups.

Group	N	Gender (male/female)	Age (years)	GCS score at admission	Hematoma volume (mL)	Time from onset to surgery (d)
ROSA group	71	28/13	57.24 ± 10.23	11.62 ± 3.25	34.26 ± 10.62	4.61 ± 1.21
Frame group	71	30/11	59.41 ± 10.01	11.45 ± 3.41	32.96 ± 10.26	4.42 ± 1.34
χ^2^/t		0.243	1.278	0.304	0.742	0.887
P		0.627	0.204	0.762	0.459	0.377

***Notes:*** ROSA: robot of stereotactic assistant; GCS: Glasgow Coma Scale.

**Table II:** Comparison in clinical indexes between two groups (*x̅*±*s*)

Group	N	Surgical duration (min)	Bleeding volume (mL)	Postoperative extubation time (d)
ROSA group	71	28.62 ± 7.89	42.65 ± 10.65	1.36 ± 0.41
Frame group	71	41.05 ± 9.46	45.64 ± 11.78	3.52 ± 0.94
t		8.502	1.586	17.747
P		0.000	0.115	0.000

The incidences of infection and postoperative rehemorrhage in the ROSA group were lower than those in the frame group (*P* < 0.05), as seen in Table-III. After treatment, TNF-α, hs-CRP and IL-6 levels in the two groups were significantly lower than those before treatment (*P* < 0.05). In the ROSA group, postoperative TNF-α, hs-CRP, IL-6 and NSE levels were significantly lower while NGF and BDNF levels were higher than those in the frame group (all *P* < 0.05) (Table-IV). No statistically significant differences were found in NSE, NGF or BDNF levels in the two groups before and after treatment (*P* < 0.05). After treatment, the NSE level was lower while NGF and BDNF levels were higher in the ROSA group compared with the frame group (*P* < 0.05), as shown in Table-V.

**Table III:** Comparison in complications between two groups [N (%)].

Group	N	Infection	Postoperative rehemorrhage
ROSA group	71	0 (0.00)	1 (1.41)
Frame group	71	9 (12.68)	11 (15.49)
χ^2^		7.592	7.373
P		0.006	0.007

**Table IV:** Comparison in inflammatory factor levels between two groups (*x̅*±*s*).

Group	N	TNF-α (ng/L)		hs-CRP (mg/L)		IL-6 (μg/L)	
		
		Before treatment	After treatment	Before treatment	After treatment	Before treatment	After treatment
ROSA group	71	50.41 ± 13.62	35.62 ± 9.74*^a^*	16.21 ± 4.62	7.20 ± 2.21*^a^*	65.42 ± 16.25	39.65 ± 9.58*^a^*
Frame group	71	48.56 ± 14.57	42.62 ± 11.10*^a^*	15.69 ± 4.74	10.56 ± 2.93*^a^*	63.79 ± 16.77	48.62 ± 12.34*^a^*
t		0.782	3.994	0.662	7.714	0.588	4.838
P		0.436	0.000	0.509	0.000	0.557	0.000

***Notes:*** Compared with before treatment,

a *P* < 0.05; TNF-α: tumor necrosis factor-α; hs-CRP: high-sensitivity C-reactive protein; IL-6: interleukin-6.

**Table V:** Comparison in neurological function indexes between two groups (*x̅*±*s*).

Group	N	NSE (U/mL)		NGF (pg/mL)		BDNF (pg/mL)	
		
		Before treatment	After treatment	Before treatment	After treatment	Before treatment	After treatment
ROSA group	71	31.75 ± 8.76	19.63 ± 5.76^a^	19.46 ± 5.13	32.39 ± 8.34^a^	14.17 ± 3.89	25.36 ± 5.29^a^
Frame group	71	30.43 ± 8.65	25.54 ± 6.88^a^	20.34 ± 5.68	25.38 ± 7.36^a^	14.54 ± 3.63	19.22 ± 4.85^a^
t		0.903	5.550	0.968	5.310	0.586	7.209
P		0.368	0.000	0.334	0.000	0.559	0.000

***Notes:*** Compared with before treatment,

a *P* < 0.05; NSE: neuron-specific enolase;

NGF: nerve growth factor; BDNF: brain-derived neurotrophic factor.

## DISCUSSION

HICH is one of the most severe complications of hypertension. Long-term hypertension can cause swelling and lipid precipitation of the vascular intimal matrix, resulting in reduced vascular elasticity, increased brittleness, fibrinoid necrosis and vitreous degeneration of the vascular wall.^6^ For HICH patients with supratentorial hematoma volume ≥ 20mL or infratentorial hematoma volume ≥ 10 mL, surgery is still the primary choice.^7^ With the development of neuroendoscopy, the application of brain microsurgery is becoming increasingly mature, and most intracranial space-occupying lesions can be removed by microsurgery.^8^ Stereotactic technology was firstly reported to be used in the treatment of HICH in 1978, with the advantages of small iatrogenic injury and no need for general anesthesia.^9^

Frame-assistant stereotactic drilling and drainage can help HICH patients rapidly remove hematoma and relieve the adverse effects caused by the decomposition products and space-occupying effect of hematoma, thus reducing intracranial pressure and avoiding the progressive development of encephaledema.^10^ ROSA is a multifunctional robot with stereotactic localization and sensing technology of six degrees of freedom (6-DOF) manipulator, which integrates multiple functions such as personalized surgical planning system, navigation, auxiliary positioning and operating system. It can be rapidly and accurately applied to the microsurgical treatment of brain diseases such as HICH and epilepsy.^11^,^12^

In our study, 142 HICH patients were divided into two groups: ROSA group was treated with stereotactic assisted robot, and frame group was treated with stereotactic assisted drilling and drainage of intracranial hematoma. The results of this study found that, compared with the traditional stereotactic frame system, ROSA adjuvant therapy for HICH has the following advantages:

### Shortened surgical duration

The ROSA system is easy to operate, and it operates according to the preset surgical plan without manual calculation, saving the installation time of components. The shortened surgical duration can reduce the exposure time of brain tissue, decrease the risk of infection and improve the long-term efficacy. Which was similar to the research results of Spyrantis A et al.^13^

### Personalized surgical approach selection

ROSA system can carry out three-dimensional (3D) reconstruction for the morphology of cerebral hematoma in HICH patients, help surgeons understand the morphological characteristics of hematoma more clearly and intuitively, and facilitate the formulation of personalized surgical plans. The selection of surgical approach is more scientific and reasonable. The inserted drainage tube can coincide with the maximum diameter of hematoma in multiple dimensions. The effect of surgical removal of hematoma is better, and the postoperative extubation time is shorter than that of patients using the stereotactic frame. For surgical approach in HICH patients using stereotactic frame system, because of the unobservable 3D morphology of hematoma, it is impossible to ensure that the drainage tube is inserted completely along the long axis of hematoma, which delays the postoperative extraction time of drainage tube and increases the risk of infection. Which was similar to the results of previous studies.^14^

High safety. ROSA system has larger surgical space and more convenient operation due to no frame interference. With a stereotactic frame system, it needs to wear a headframe before surgery and then conduct CT scanning, which is not conducive to emergency surgery. The dural incision is smaller than that of the stereotactic frame system, which reduces the risk of postoperative pneumocranium, accelerates postoperative recovery and decreases complications. This was similar to the results of Alan N et al.^15^

Previous studies on ROSA mainly focused on its short-term and long-term efficacy, but its impacts on body inflammatory state and neurological function were rarely reported.^16^ Relevant studies have pointed out that the inflammatory reaction between the hematoma site of intracerebral hemorrhage and its surrounding tissues in HICH patients aggravates brain tissue injury and deterioration of neurological function.^17^ TNF-α is a systemic inflammatory factor mainly secreted by macrophages, which can mediate immune injury after cerebral ischemia.^18^ CRP is an acute-phase protein during tissue injury, and its sensitivity is greatly improved by hs-CRP detection.^19^ As a chemokine of the body, IL-6 can enhance the scavenging effect of natural killer cells, increase the production of free radicals and induce the death of neurons.^20^ The results of this study showed that, compared with the traditional 3D frame system, the postoperative serum TNF-α, hs-CRP and IL-6 levels were lower in patients treated with ROSA. The reason may be that the HICH patients treated with ROSA have less intraoperative tissue injury, short incision exposure time and less surgical stress, so the inflammatory level is low. The recovery of neurological function of the brain is of great significance for the prognosis of HICH patients.^21^ NSE is an enolase widely existing in nerves. When cerebral ischemia-hypoxia occurs, neurons rupture and the NSE level in the peripheral blood increases significantly.^22^ NGF and BDNF are both cytokines with neuronal nutrition and repairing effects. When brain tissue injury occurs, the expressions of NGF and BDNF are up-regulated to repair neurons.^23^,^24^ Our results revealed that the serum NGF and BDNF increased significantly while the NSE level decreased in the two groups after surgery, indicating that surgical treatment can reduce intracranial pressure and alleviate brain tissue injury while removing the hematoma. The improvement of serum neurological function factors in the ROSA group was significantly superior to that in the control group, which may be related to less tissue injury and shorter drainage time in the treatment of HICH by ROSA, which is helpful to the recovery of neurons.

### Limitations of this study

The number of subjects included in this study was limited, so the conclusions drawn may not be very convincing. In addition, we only analyzed and discussed the cases included in our hospital, which may not be representative enough. We look forward to a multi-center study in the future to reach more comprehensive conclusions.

## CONCLUSION

Compared with frame-assisted stereotactic drilling and drainage for intracerebral hematoma, ROSA in HICH treatment shortens the surgical duration and postoperative extubation time, reduces the risks of infection and rehemorrhage, and decreases inflammatory level, which is helpful for the recovery of neurological function.

### Authors’ Contributions:

**LL** & **XL:** Designed this study , prepared this manuscript, are responsible and accountable for the accuracy, integrity of the work.

**HD & XG:** Collected and analyzed clinical data.

**GW:** Significantly revised this manuscript.
